# Metabolic pathways in tropical dicotyledonous albuminous seeds: *Coffea arabica* as a case study

**DOI:** 10.1111/j.1469-8137.2008.02742.x

**Published:** 2009-04

**Authors:** Thierry Joët, Andréina Laffargue, Jordi Salmona, Sylvie Doulbeau, Frédéric Descroix, Benoît Bertrand, Alexandre de Kochko, Stéphane Dussert

**Affiliations:** 1 IRD, UMR DIA-PC, Pôle de Protection des Plantes97410, Saint Pierre, La Réunion, France; 2 IRD, UMR DIA-PCBP 64501, 34394 Montpellier, France; 3 CIRAD, UMR QualiSud97410, Saint Pierre, La Réunion, France; 4 CIRAD, UMR RPBBP 64501, 34394 Montpellier, France

**Keywords:** albuminous seed development, *Coffea arabica* (coffee), gene profiling, metabolic pathways

## Abstract

- The genomic era facilitates the understanding of how transcriptional networks are interconnected to program seed development and filling. However, to date, little information is available regarding dicot seeds with a transient perisperm and a persistent, copious endosperm. *Coffea arabica* is the subject of increasing genomic research and is a model for nonorthodox albuminous dicot seeds of tropical origin.
- The aim of this study was to reconstruct the metabolic pathways involved in the biosynthesis of the main coffee seed storage compounds, namely cell wall polysaccharides, triacylglycerols, sucrose, and chlorogenic acids. For this purpose, we integrated transcriptomic and metabolite analyses, combining real-time RT-PCR performed on 137 selected genes (of which 79 were uncharacterized in *Coffea*) and metabolite profiling.
- Our map-drawing approach derived from model plants enabled us to propose a rationale for the peculiar traits of the coffee endosperm, such as its unusual fatty acid composition, remarkable accumulation of chlorogenic acid and cell wall polysaccharides.
- Comparison with the developmental features of exalbuminous seeds described in the literature revealed that the two seed types share important regulatory mechanisms for reserve biosynthesis, independent of the origin and ploidy level of the storage tissue.

## Introduction

The endosperm of angiosperm seeds originates from the fusion in the central cell of the embryo sac of the two female polar nuclei with one of the two male gametes delivered by the pollen tube, the second fertilization generating the embryo. The relative contribution of the endosperm to the final mass of the mature seed varies greatly depending on the species. It ranges from nonexistent (e.g. soybean, rape, pea), to one or a few peripheral layers of cells surrounding a reserve-containing embryo (e.g. *Arabidopsis thaliana*, *Medicago truncatula*, tobacco), to constituting most of the seed volume (e.g. barley, oil palm, castor bean and coffee) (Forbis*et al.*, 2002; Finch-Savage & Leubner-Metzger, 2006). In the latter case (albuminous seeds), triploid endosperm is always the major storage tissue, but its structure may differ considerably. In the caryopsis of cereals, the endosperm mainly consists of dead storage tissue, the starchy endosperm, surrounded by the living aleurone layer. By contrast, in albuminous dicots, the endosperm is composed solely of uniform living reserve cells. A tiny embryo encapsulated in a copious endosperm, as in coffee, characterizes the plesiomorphic state in seed plants (Forbis*et al.*, 2002). In this seed category, endosperm cells undergo programmed cell death only after reserves have been mobilized during seedling growth. Thanks to the emergence of transcriptomic, proteomic and metabolomic techniques, global analysis of seed filling has been an active area of research in the last 10 yr. Most efforts have targeted crop and model species, such as barley, rape, soybean, *Arabidopsis thaliana* and *Medicago truncatula* (Ruuska*et al.*, 2002; Sreenivasulu*et al.*, 2004; Hajduch*et al.*, 2005, 2006; Spencer*et al.*, 2007; Gallardo*et al.*, 2007; Santos-Mendoza*et al.*, 2008). A comprehensive survey of the literature revealed that most of our current knowledge of these processes concerns exalbuminous seeds, that is, seeds containing a thin residual endosperm layer, and caryopses. Very few studies have focused on albuminous seeds, such as those of castor bean or coffee.

The coffee endosperm (approx. 99% of the mature seed mass) contains all the necessary precursors to generate coffee aromas during roasting. The major storage compounds of mature seeds of *Coffea arabica* are cell wall polysaccharides (CWP, 48–60% DM), mainly galactomannans and arabinogalactan-proteins (Redgwell *et al*., 2002, 2003), lipids (13–17% DM), mainly triacylglycerols (TAG) (Speer & Kölling-Speer, 2006), proteins (11–15% DM, Clifford, 1987), sucrose (7–11% DM, Campa*et al.*, 2004) and chlorogenic acids (CGA) (5–8% DM, Farah & Donangelo, 2006). Each of these major storage compounds plays several crucial roles in the complex roasting chemistry (Flament, 2002). For example, proteins and amino acids are essential for conversion of reducing sugars (which result from the degradation of sucrose and CWP) into aroma precursors through Maillard reactions (De Maria*et al.*, 1996); TAG is the major aroma carrier in the roasted bean (Petracco, 2005); their fatty acid (FA) composition determines the generation of thermally induced oxidation products, in particular aldehydes, which react readily with Maillard intermediates, giving rise to additional aroma compounds (Flament, 2002); CGA and caffeine, are responsible for bitterness (Leloup*et al.*, 1995). In addition, the coffee seed presents several distinctive characteristics that are not encountered in seeds of model plants. Like some leguminous seeds that retain an endosperm in the mature state (e.g. *Lotus japonicus*) and some Arecaceae (e.g. date palm), the storage polysaccharides of the coffee seed are located in the cell wall. Among these plants, coffee stands out because the galactose/mannose ratio in galactomannans is very low (Redgwell*et al.*, 2003; Reid*et al.*, 2003; Edwards*et al.*, 2004). Like many other tropical oily seeds, such as oil palm, cocoa, shea, and coconut, coffee seeds contain a very high proportion of saturated fatty acids (*c*. 40%) (Speer & Kölling-Speer, 2006). However, in contrast to other tropical plants, coffee seeds have the very unusual characteristic that the remaining fatty acids are not monounsaturated but polyunsaturated. Another peculiar feature of coffee seeds is the fact that they accumulate considerable amounts of chlorogenic acids, which are mobilized during germination for seedling lignin metabolism (Aerts & Baumann, 1994).

For coffee breeding, it is thus important to have an overview of the metabolic networks in the developing endosperm. This would enable a better understanding of the storage process, elucidate the transient role of perisperm – a diploid maternal tissue that originates from the nucellus – in the synthesis and transport of storage products in early developmental stages, and, finally, help identify candidate genes and molecular markers associated with target biochemical traits. For this purpose, a fine survey of gene expression and metabolite profiles is required throughout seed development and maturation. Despite the recent release of several expressed sequence tag (EST) databases (Lin*et al.*, 2005), very few studies have simultaneously dealt with gene expression and metabolite profiling during coffee seed development. To date, only a few genes (15) involved in sucrose and CGA metabolism have been investigated using this approach (Geromel*et al.*, 2006; Lepelley*et al.*, 2007; Privat*et al.*, 2008). There was therefore an urgent need for a more global approach. To this end, we recently performed a transcriptomic study on developing *Coffea arabica* seeds using combined cDNA array and real-time RT-PCR (Salmona*et al.*, 2008). This study, which enabled us to decipher the major transcriptional re-programming events between early cell division and elongation, storage and maturation phases, was partly based on genes involved in the metabolism of storage compounds. However, no particular pathway was extensively analyzed and, moreover, a survey of gene expression profiles alone is not sufficient to obtain a clear picture of the metabolic networks that exist during seed development.

The aim of the present work was, for the first time, to piece together the main metabolic pathways in a developing transient perisperm-persistent living endosperm seed using *Coffea* as a model. The publicly available *Coffea* EST databases were exploited to design real-time RT-PCR primers for each gene suspected of being involved in the biosynthetic pathways of the metabolites under study. We focused on TAG, CGA and sugar metabolism. The other major class of storage compounds (proteins) was not investigated since in this case a proteomic approach seemed to be more appropriate and has already been partially accomplished for the major 11S-type albumin (Rogers*et al.*, 1999a). Relative transcript patterns for 79 nonredundant genes are reported here for the first time. For an integral view of key biosynthetic pathways, we also included an additional 58 recently published gene expression profiles (Salmona*et al.*, 2008).

## Materials and Methods

### Experimental site and plant material

**Fig. 1:**
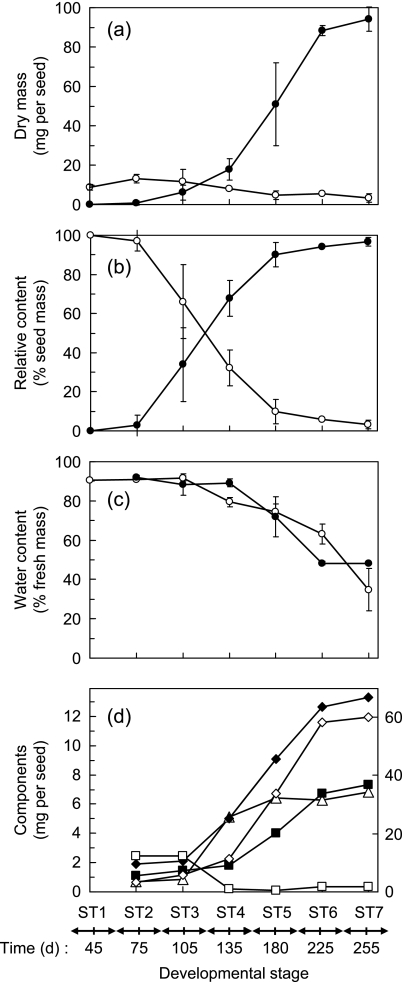
Characterization of developing coffee (*Coffea arabica*) seeds. Sampling was carried out at seven phenological stages from aqueous perisperm to ripe endosperm. Changes in perisperm (open circles) and endosperm (closed circles) dry mass (a), relative proportion (b) and water content (c). Error bars indicate ± SD of repetitions (*n* = 3). Changes in seed content (d) in lipids (closed diamonds), chlorogenic acids (open triangles), sucrose (closed squares), hexoses (open squares), and cell-wall polysaccharides (open diamonds). The secondary *y* axis is for cell wall polysaccharides.

Experiments were performed on seeds of *Coffea arabica* L. cv. ‘Laurina’. Developing seeds were harvested from plants grown at Grand Tampon, Reunion Island, France (21.16°S, 55.33°E, 1015 m asl) on an Andosol soil, with an average temperature of 18.5°C and a mean annual rainfall of 1300 mm. Coffee trees were planted in 2002 without shade and were in their second (2005) production cycle. Plant spacing was 2 m between rows and 1 m within rows. For each of the seven developmental stages studied (Fig. 1; Salmona*et al.*, 2008), three independent biological samples (pools of *c*. 200 seeds) were collected on 20 trees randomly selected in the experimental plot. After cross-section, the seed was separated from the pericarp and locules and immediately frozen in liquid nitrogen and stored at −80°C before RNA extraction. For chemical analysis, seed tissues were freeze-dried and ground to a fine powder using an analytical grinder (IKA A10, Staufen, Germany). Powdered seed tissue was stored over silica-gel in a hermetic plastic box at −20°C until analysis. For each developmental stage, four to five fresh fruits were dissected in order to measure separately perisperm and endosperm masses. The dry mass was measured after 1 d of drying in an oven at 103°C.

### Real-time RT-PCR assays

Total RNA extraction and real-time RT-PCR assays were performed as previously described (Salmona*et al.*, 2008). Roughly, first strand cDNA synthesis was performed using the ImProm-II reverse transcription system (Promega, Madison, WI, USA) using 1 µg of total RNA (treated with RNase-free DNase I, Promega) as a template. Polymerase chain reactions were carried out in an optical 96-well plate with an ABI Prism 7000 sequence detection system (Applied Biosystems, Foster City, CA, USA), using SYBR Green to monitor dsDNA synthesis. Data were analyzed using the SDS 2.1 software (Applied Biosystems) to determine cycle threshold (*Ct*) values. PCR efficiency (*E*) was estimated for each gene with LinReg software, which uses absolute fluorescence data captured during the exponential phase of amplification of each reaction using the equation (1 + *E*) = 10^(−1/slope)^ (Ramakers*et al.*, 2003). Efficiency values were taken into account in all subsequent calculations. Normalization was processed by using the mean expression (*Ct* value) for all genes under study as the inter-PCR run effect was considered negligible, as tested by the use of an internal reference (ubiquitin) in each plate. Relative expression rate of target gene transcripts were then calculated (Supporting Information, Table S2) using the following formula:

$${\text{Fold change}=(1+E)}^{-\Delta \Delta Ct}$$

where

$${\Delta Ct=Ct}_{\text{target gene}}{–Ct}_{\text{mean of all genes}}$$

and

$${\Delta \Delta Ct= \Delta Ct}_{\text{stage of interest}}{- \Delta Ct}_{\text{stage of maximal expression}}$$

The specificity of the PCR products generated for each set of primers (Table S3) was tested by agarose gel electrophoresis (Fig. S1), and sequencing of PCR products.

The Coffee EST database used in this project (SOL genomics networks; http://sgn.cornell.edu/) currently contains > 15 000 unigenes (deduced from 57 000 ESTs from *C. arabica* and *C. canephora*; Lin *et al.*, 2005). For FA and TAG biosynthesis, we performed an exhaustive search for orthologs with TBLASTN, using sequences from the Arabidopsis Lipid Gene Database as templates (Beisson *et al.*, 2003). In the same way, for the phenylpropanoid pathway, we used the well-characterized gene repertoire for lignification in *Arabidopsis thaliana* (Raes *et al*., 2003; Kim *et al*., 2004; Costa *et al*., 2005) to find orthologs. By contrast, metabolic pathways involved in CWP biosynthesis, especially galactomannans and pectins, have not been so comprehensively described in model plants. Only a few genes were cloned and the corresponding enzymes functionally characterized. Since it is hazardous to deduce functions from weak sequence homologies, we only retained coffee sequences highly homologous to genes functionally characterized in model plants (Edwards *et al.*, 1999; Seitz *et al.*, 2000; Sprenger & Keller, 2000; Hegeman*et al.*, 2001; Reid *et al.*, 2003; Dhugga*et al.*, 2004; Gu & Bar-Peled, 2004; Kanter *et al.*, 2005; Marraccini *et al.*, 2005; Nguema-Ona *et al.*, 2006; Sterling *et al.*, 2006).

### Lipid, sugar and chlorogenic acid determination

**Fig. 2:**
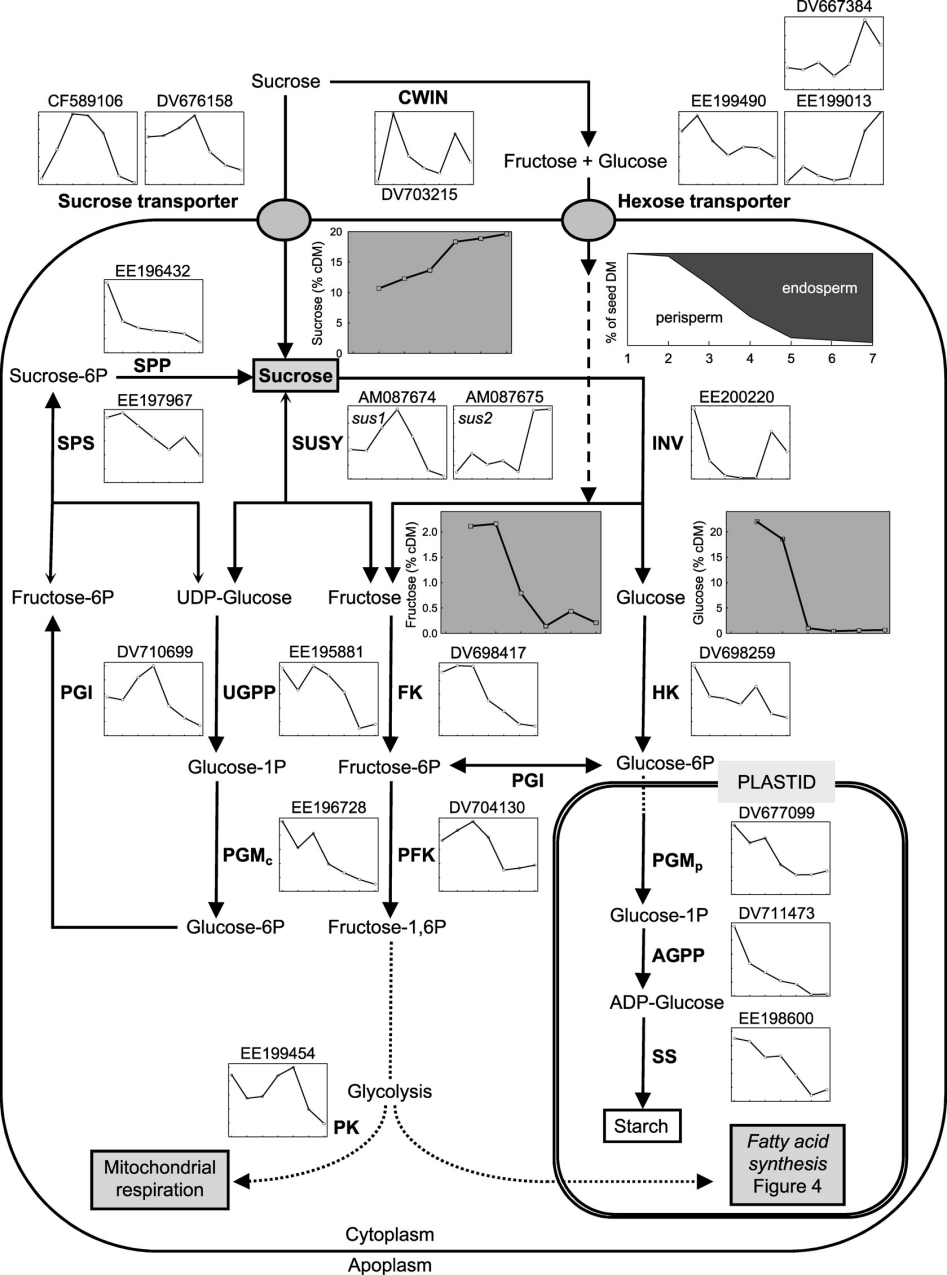
Mapping of transcripts and metabolites associated with sucrose metabolism throughout coffee (*Coffea arabica*) seed development. Metabolite profiles are in gray boxes and gene expression profiles are in white boxes. Dashed lines indicate multistep pathways. AGPP, ADP-glucose pyrophosphorylase; CWIN, cell wall invertase; FK, fructokinase; HK, hexokinase; INV, invertase; PFK, phosphofructokinase, PGMc and PGMp, cytosolic and plastidial phosphoglucomutase, respectively; PGI, phosphoglucose isomerase; PK, pyruvate kinase; SPP, sucrose phosphate phosphatase; SPS, sucrose phosphate synthase; SS, starch synthase; SUSY, sucrose synthase; UGPP, UDP-glucose pyrophosphorylase.

**Fig. 3:**
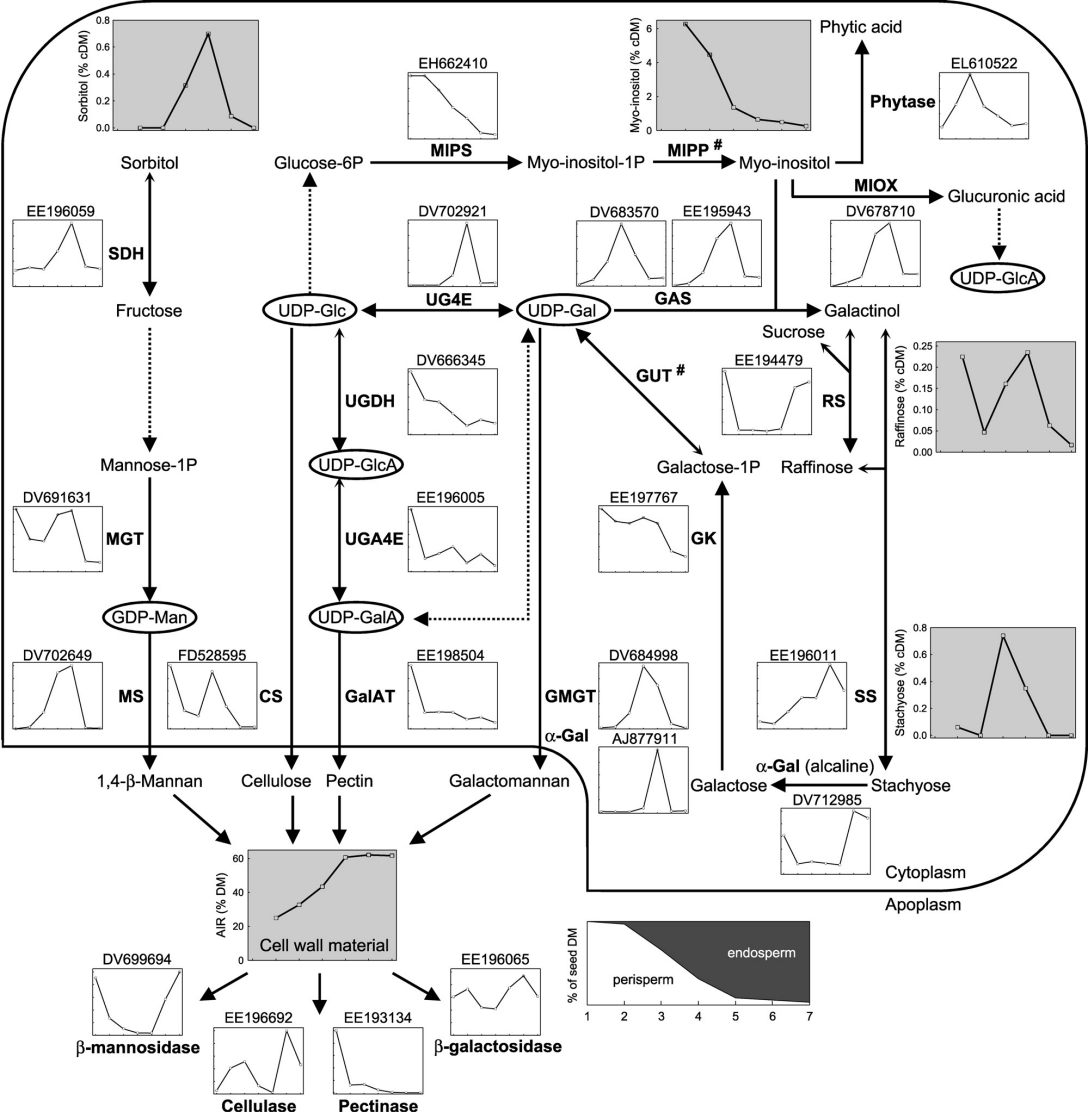
Carbohydrate metabolism in the developing coffee (*Coffea arabica*) seed. A schematic diagram of the interactions involved in the interconversion of carbon from fructose and glucose into either sorbitol, phytic acid, galactose, raffinose, stachyose and cell wall polymers such as cellulose, pectin and mannan. Metabolite profiles are in gray boxes and gene expression profiles are in white boxes. Dashed lines indicate multistep pathways. Direct building blocks for CWP (cell wall polysaccharides) are circled. CS, cellulose synthase; GalAT, galacturonosyl transferase; α-Gal, alpha-galactosidase; GAS, galactinol synthase; GK, galactinol kinase; GMGT, galactomannan galactosyltransferase; GUT, Glc uridyltransferase; MGT, mannose-1P guanyltransferase; MIOX, myo-inositol oxygenase; MIP, myo-inositol 1-phosphate; MIPP, myo-inositol 1-phosphate phosphatase; MIPS, myo-inositol 1-phosphate synthase; MS, beta 1,4-mannan synthase; RS, raffinose synthase; SDH, sorbitol dehydrogenase; SS, stachyose synthase; UDP-Gal, UDP-galactose; UDP-GalA, UDP-galacturonic acid; UG4E, UDP-glucose 4′-epimerase; UDP-Glc, UDP-glucose; UDP-GlcA, UDP-glucuronic acid; UGA4E, UDP-glucuronic acid 4′-epimerase; UGDH, UDP-Glc dehydrogenase. #, no candidate gene found in *Coffea* EST databases.

**Fig. 4:**
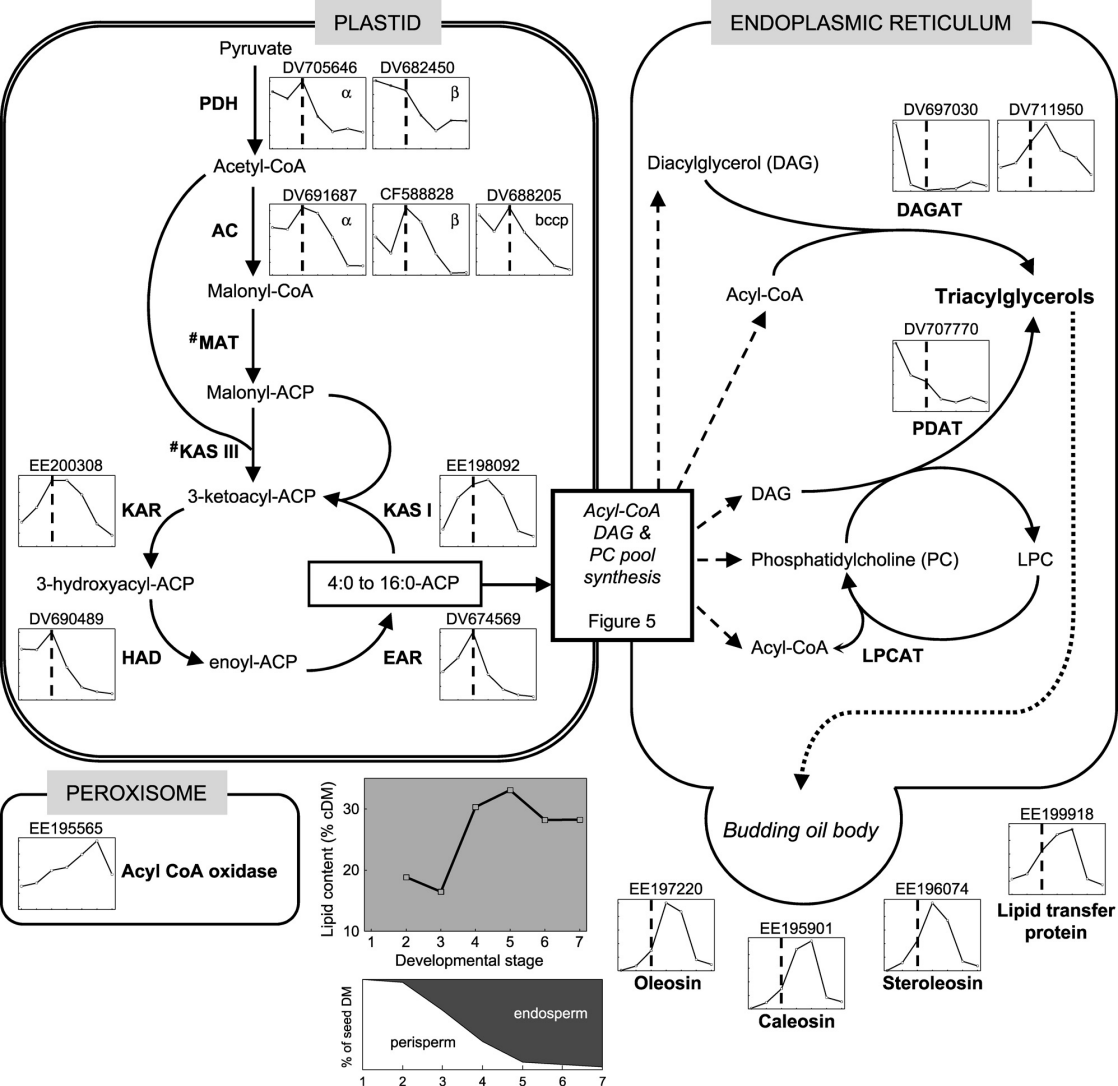
Fatty acid and triacylglycerol synthesis in the developing coffee (*Coffea arabica*) seed. Metabolite profiles are in grAy boxes and gene expression profiles are in white boxes. The peak of transcriptional activity for fatty acid synthesis activity genes (stage 3) is marked by a dotted line. AC, acetyl-CoA carboxylase; DAG, diacylglycerol; DAGAT, diacylglycerol acyltransferase; EAR, enoyl-ACP reductase; HAD, hydroxyacyl-ACP dehydrase; KAR, ketoacyl-ACP reductase; KAS, ketoacyl-ACP synthase; LPC, lysophosphatidylcholine; LPCAT, lysophosphatidylcholine acyltransferase; MAT, malonyl-CoA ACP transacylase; PC, phosphatidylcholine; PDAT, phospholipid diacylglycerol acyltransferase; PDH, pyruvate dehydrogenase. #, no candidate gene found in *Coffea* expressed sequence tag (EST) databases.

**Fig. 5:**
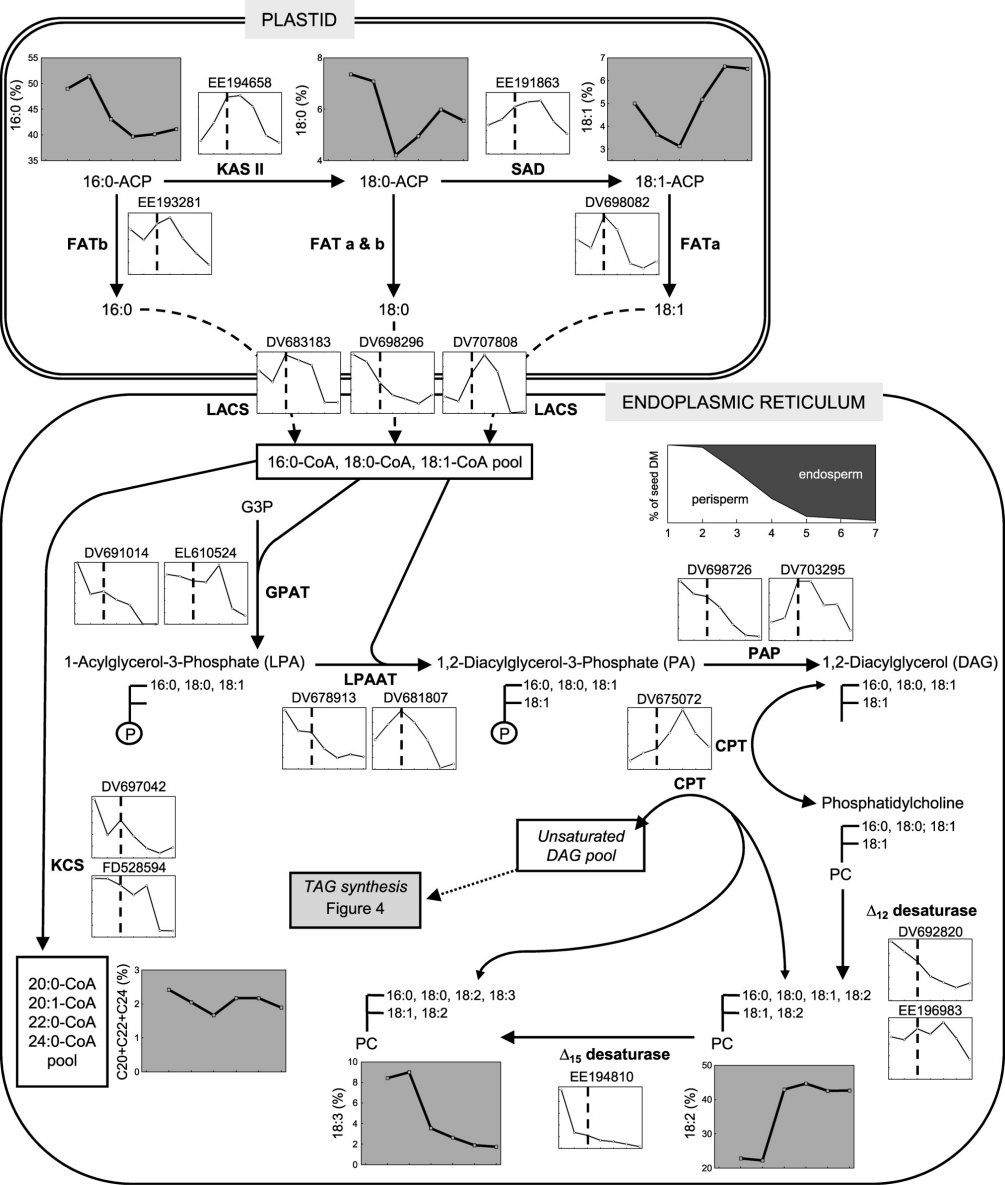
Acyl CoA, diacylglycerol and phosphatidylcholine pool synthesis in the developing coffee (*Coffea arabica*) seed. Metabolite profiles are in gray boxes and gene expression profiles are in white boxes. The peak of transcriptional activity for fatty acid synthesis genes (stage 3) is marked by a dotted line. CPT, diacylglycerol cholinephosphotransferase; FAT, acyl-ACP thioesterase; GPAT, glycerol-phosphate acyltransferase; KAS, ketoacyl-ACP synthase; KCS, beta ketoacyl-CoA synthase; LACS, long-chain acyl-CoA synthetase; LPAAT, acylglycerol-phosphate acyltransferase; PAP, phosphatidate phosphatase; PC, phosphatidylcholine; SAD, stearoyl-ACP desaturase.

**Fig. 6:**
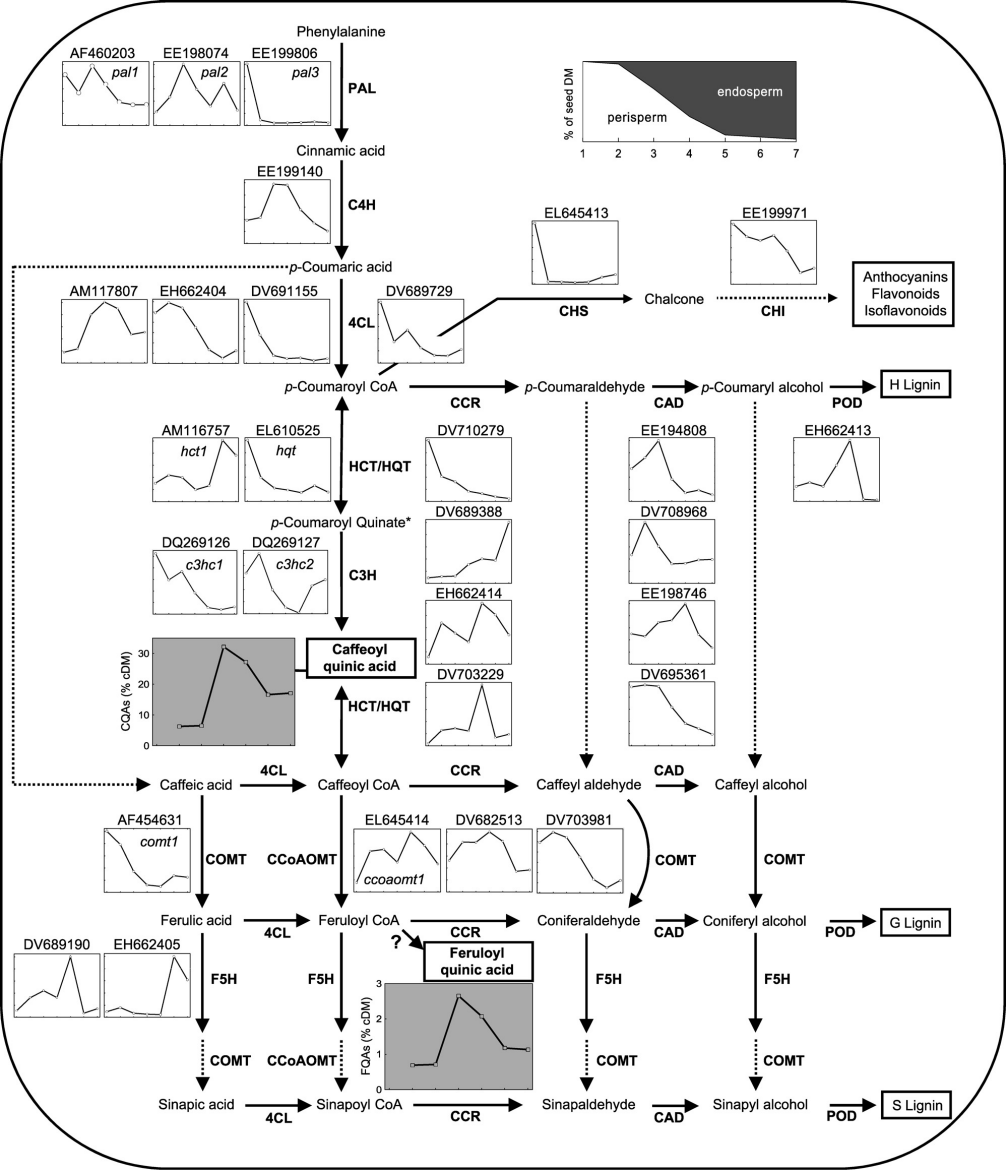
Changing patterns of gene expression (white boxes) associated with chlorogenic acids and with monolignols biosynthetic pathway in the developing coffee (*Coffea arabica*) seed. Caffeoyl quinic and feruloyl quinic acids (gray boxes) are intermediate metabolites leading to G and S monolignols. Dashed lines indicate multistep pathways or uncharacterized enzymes. C3H, p-coumarate 3-hydroxylase; C4H, trans-cinnamate 4-hydroxylase; CAD, cinnamyl alcohol dehydrogenase; CCoAOMT, caffeoyl-CoA 3-O-methyltransferase; CHS, chalcone synthase; CHI, chalcone isomerase; 4CL, 4-coumarate:CoA ligase; CCR, cinnamoyl-CoA reductase; COMT, caffeic acid O-methyltransferase; CQAs, caffeoyl quinic acids; F5H, ferulate 5-hydroxylase; FQAs, feruloyl quinic acids; HCT, hydroxycinnamoyl-CoA:shikimate/quinate hydroxycinnamoyl transferase; HQT, hydroxycinnamoyl-CoA quinate hydroxycinnamoyl transferase; PAL, phenylalanine ammonia lyase; POD, secretory peroxidase. *, p-coumaroyl quinate or p-coumaroyl shikimate.

Total lipids were extracted from 300 mg samples of freeze-dried powder using a modified Folch method (Folch*et al.*, 1957) as described in Laffargue *et al*. (2007). Fatty acid methyl esters (FAMEs) were prepared according to the ISO-5509 standard, and GC analyses of FAMEs were performed as previously described (Laffargue*et al.*, 2007). Sugars were extracted and measured by high-performance anion exchange chromatography coupled with pulsed amperometric detection (Dionex Chromatography Co., Sunnyvale, CA, USA) as described elsewhere (Dussert*et al.*, 2006). The seed content in CWP was estimated by measuring the defatted alcohol insoluble residue (DAIR) using the method developed by Redgwell *et al*. (2003). Chlorogenic acids were extracted as described in Bertrand *et al.* 2003 and then analyzed using the HPLC conditions employed previously (Bertrand*et al.*, 2008). All metabolites were analyzed in triplicate (from three different extractions) using a completely random experimental design. Estimation of CWP throughout seed development allowed us to report lipids, free sugars and CGA contents on a corrected dry mass basis (cDM = DM/(100 – DAIR content) × 100, Figs 2–6) giving a better estimate of these compounds at the intracellular level.

## Results

### Development of the coffee seed and accumulation of reserves

Under the field conditions used in this study, fruits of *C. arabica* completed their maturation in approx. 260 d. Until 75 d after flowering (DAF) (stage 2), the perisperm exhibited a significant increase in dry mass (DM) (Fig. 1a), which comprised the major part of the seed until stage 3 (Fig. 1b). Afterwards, the endosperm developed rapidly and replaced the perisperm in the locule (Fig. 1a). Endosperm DM increased considerably until 225 DAF (stage 6, ripening stage when the pericarp of the cherry fruits turns yellow), then remained fairly constant during the last month of development that leads to the mature stage when the cherry fruit with the pericarp turns red and purple (Fig. 1a). At this stage, the endosperm represented > 98% of the mature seed mass (stage 7, Fig. 1b). Unlike in orthodox seeds, coffee seed development does not end with a significant drop in water content (i.e. a desiccation stage *sensu stricto*) (Fig. 1c). Instead, the endosperm exhibited a progressive decrease in water content throughout its development, but its final water content did not drop below 50%. Lipids and CGA started to accumulate as soon as the endosperm began to develop (from stage 3, Fig. 1d, Table S1). However, in contrast to lipids whose pattern reflected that of endosperm DM and accumulated until stage 6 (Fig.1a–d), CGA deposition ended early (i.e. by stage 4). Hexoses were the main free sugars during stages 2–3 when the perisperm predominated. Both CWP and sucrose began to accumulate by stage 4, that is, later than lipids and CGA (Fig. 1d). However, like lipids, storage of all sugar compounds ended by stage 6.

### Hexose permeation pathways and starch metabolism in the transient perisperm

One of the most noticeable findings arising from sugar profiling during coffee seed development was the very high proportion of glucose, myo-inositol and fructose at early stages, when the transient perisperm predominated (16.5, 4.7 and 1.6% DM, respectively, at stage 2, Table S1). Their content dropped abruptly as soon as the endosperm started expanding (by stage 4), whereas sucrose content remained fairly constant during the perisperm-endosperm transition (7–8% DM). Assuming that sucrose is the major carbohydrate transported in the phloem of coffee trees (Geromel*et al.*, 2006), one can undoubtedly presume that the high concentrations of glucose and fructose observed in the perisperm are caused by enhanced sucrose catabolism, which could occur either in the apoplasm, through the action of cell wall invertase, or intracellularly via invertase and/or sucrose synthase activities. The low expression of sucrose synthase genes (*sus1* and *sus2*) in early developmental stages suggests an important role for invertases (Fig. 2). This hypothesis is strengthened by the high levels of expression of cell wall and vacuolar invertase genes (DV703215 and EE200220, respectively) observed at early stages. In order to be effective, cell wall invertase activity has to be coupled to a plasma membrane transporter for intracellular hexose uptake. We identified three putative plasma membrane hexose transporters, whose transcript abundance varied considerably over the course of seed development. Among them, only one gene (EE199490) displayed maximal expression in early developmental stages and thus represents a good candidate for functional characterization. Once present in the cytosol, fructose and glucose are further converted into their corresponding hexose phosphates for metabolic activation. Interestingly, the pattern of expression of a fructokinase gene perfectly matched that of fructose content. To a lesser extent, the drop in glucose was in line with a progressive decline in hexokinase transcript abundance. The resulting hexose phosphates can be further metabolized through glycolysis, starch synthesis and lipid synthesis. Transcripts encoding plastid enzymes involved in starch biosynthesis, such as phosphoglucomutase, glucose-1P pyrophosphorylase and starch synthase, all accumulate in early developmental stages and then progressively decline during seed development. Our data clearly revealed active starch metabolism in the transient perisperm of the coffee seed.

### Sucrose permeation pathways during the storage process

In contrast to hexoses, sucrose is a nonnegligible component of storage compounds (7.5% DM in mature seeds; Table S1) and represents a source of energy that is easily mobilized during germination. There was a significant increase in sucrose content during the mid-stages of endosperm development (between stages 4 and 6) (Figs 1d, 2). Two main mechanisms may explain this increase. On the one hand, sucrose may be directly loaded intracellularly across the plasma membrane by sucrose transporters. Interestingly, the two potential sucrose/H^+^ cotransporter genes we identified both showed maximal expression at stages 3–4 when the endosperm is developing rapidly and starts to store TAG and CGA (from stage 3 on) and CWP (from stage 4 on). This hypothesis for increased sucrose import during the storage phase was corroborated by the expression profile of the sucrose synthase gene *sus1*, which is most likely involved in sucrose catabolism and was closely associated with the expression profiles of sucrose transporters. On the other hand, sucrose could also be synthesized *de novo* via the coupled enzyme activities of sucrose phosphate synthase (SPS) and sucrose phosphate phosphatase (SPP). However, the two corresponding genes were expressed at maximal levels in the transient perisperm and not at stages when sucrose content increased. Consequently, the present profiling data strongly suggest that the mid-stage increase in sucrose content is controlled at the transcriptional level through enhanced expression of sucrose transporters.

### Fine transcriptional tuning during CWP synthesis: balance between genes involved in the last polymerization steps and catabolism

Hexoses resulting from sucrose cleavage are diverted to different cellular and biosynthetic functions. Among them, biosynthesis of CWP is of particular importance in coffee, since they increased dramatically when the endosperm was undergoing rapid development (between stages 3 and 6) to reach 60% DM by stage 6 (Figs 1d, 3). Key genes involved in the final polymerization steps all exhibited a transcription peak concomitant with the onset of CWP accumulation (Fig. 3), suggesting that CWP metabolism is mostly controlled at the transcriptional level. They include a cellulose synthase (CS) subunit gene and genes encoding enzymes directly involved in 1,4-β-mannan synthesis such as mannose-1P guanyltransferase (MGT) and β-1,4 mannan synthase (MS), which can be used as a signature of hemicellulosic polysaccharide biosynthesis. They also comprise genes involved in galactose-to-mannan branching responsible for galactomannan structure (a backbone of β-1,4 mannose residues with varying degrees of α-1,6 galactose substitutions), such as galactomannan galactosyltransferase (GMGT) and α-galactosidase. The transcriptional control of CWP biosynthesis in the developing coffee endosperm is also revealed by the shutdown of genes dedicated to CWP hydrolysis, such as genes encoding β-mannosidase, cellulase and, to a lesser extent, β-galactosidase, during the CWP accumulation stage.

### Supplying CWP building blocks: evidence for transient storage forms of galactose and for a functional myo-inositol oxidation pathway

Among potential precursors of CWP, myo-inositol – a major precursor of phytic acid, glucuronic acid, and galactinol – caught our attention since it was present in substantial amounts in the transient perisperm (Fig. 3). Its decline during seed development slightly preceded the drop in myo-inositol phosphate synthase (MIPS) gene transcription that encodes the key enzyme of myo-inositol biosynthesis. By contrast, the drop in myo-inositol was closely associated with the transient expression peak of phytase, encoding an enzyme directly using myo-inositol as the substrate for phytic acid synthesis. High phytase activity during this period could explain the delay between myo-inositol drop and repression of its biosynthetic genes. However, myo-inositol could also be oxidized to glucuronic acid via the myo-inositol oxygenation pathway. The myo-inositol oxygenase (MIOX) gene, which encodes a key enzyme of this pathway, showed a strong peak of transcription by stages 4–5, corresponding perfectly to cell wall thickening during endosperm development. Alternatively, UDP-glucuronic acid may be supplied through UDP-glucose dehydrogenase (UGDH) activity (Fig. 3). In the developing coffee seed, our results suggest temporal sharing of tasks by the two pathways. Indeed, in contrast to the *MIOX*gene, which showed maximal expression at mid-stages, *UGDH*and the genes for subsequent enzymes leading to pectin synthesis (i.e. UDP-glucuronic acid 4′-epimerase and galacturonosyltransferase (GalAT)) were only expressed at early stages, suggesting they play an important role in the intense cell division activity occurring in the perisperm tissue.

Besides, myo-inositol may also be diverted towards raffinose family oligosaccharides (RFO) since, together with galactose, myo-inositol is a component of galactinol. RFO, mostly raffinose and stachyose, accumulated only transiently in the developing endosperm of coffee, reaching 0.2 and 0.8% cDM, respectively, at stages 4–5 (Table S1, Fig. 3). This transient accumulation of RFO was closely associated with several genes linked to galactose metabolism that peaked at the same stages, such as genes encoding UDP-glucose 4′-epimerase, galactokinase, α-galactosidase and a protein similar to a fungal galactose oxidase. As galactose is relatively toxic in a free form (free galactose was not detected), we hypothesize that it is transiently stored in other forms (RFO) before its incorporation in galactomannans.

### The transient perisperm and the developing endosperm employ different transcriptional programs towards ER lipid biosynthesis reactions

One of the immediate findings arising from our investigations is that two paralog genes exhibiting very distinctive expression patterns were identified in most steps that occur in the ER (Figs 4, 5). Conversely, only one gene was found to be involved in the reactions that take place in the plastid. In ER reactions showing two candidate genes, one gene was assigned to lipid synthesis in the perisperm, since its transcript profile showed maximal expression at stage 1, followed by a progressive decline throughout seed development (e.g. DV697030 for DAGAT (Fig. 4)), while the other gene exhibited maximal expression at mid-stages and was consequently assigned to lipid synthesis in the endosperm (e.g. DV711950 for DAGAT (Fig. 4)). The ER-located reactions for which a perisperm form and an endosperm form could be distinguished include the enzymes involved in the sequential acylation on the *sn*-1, 2 and 3 positions of the glycerol backbone (i.e. GPAT, LPAAT, PAP and DAGAT), the desaturation at the Δ_12_ position of the acyl chain, and the synthesis of long-chain fatty acids (Figs 4, 5). In contrast to reactions occurring in the ER, we identified only one candidate gene for each plastidial enzyme studied and their transcription profiles all exhibited high expression at stage 1 and stages 3–4 (Figs 4, 5), confirming the fact that the same gene was expressed in the perisperm and the endosperm at each step in the formation of the acyl chain. Another notable mode of tissue-specific expression concerned Δ_15_ desaturase for which no paralog gene specifically expressed in the endosperm could be found (Fig. 5). Accordingly, an abrupt drop in the percentage of linolenic acid percentage occurred by stages 3–4 (Fig. 5, Table S1).

### PC-DAG interconversion as the major route for TAG enrichment in linoleic acid

In the classical Kennedy pathway of TAG formation, the third acylation is catalyzed by DAGAT using an acyl-CoA from the ER pool (Fig. 4). Since acyl-CoAs resulting from FA synthesis are saturated or monounsaturated, high 18:2 percentages in TAG, such as in the mature coffee endosperm, imply the involvement of a complementary pathway. Diacylglycerols (DAG) formed through the Kennedy pathway may be converted to PC through CPT activity (Fig. 5). Oleate esterified to the resulting PC could then be desaturated to 18:2 by the Δ_12_ desaturase. The first possible route for the incorporation of 18:2 in TAG consists in the transfer of linoleoyl from PC to DAG by the PDAT enzyme to form lyso-PC and TAG. A *PDAT* gene (DV707770), highly homologous to the Arabidopsis *PDAT* gene At5g13640, was highly expressed at stage 1, and was then progressively shut down during coffee seed development (Fig. 5). This route could therefore contribute significantly to the enrichment of TAG in 18:2 in the perisperm but not in the endosperm. A second route involves the reversibility of the reaction catalyzed by CPT. Linoleate-containing PC can therefore be converted into DAG, which, in turn, enrich TAG in PUFA through the Kennedy pathway (Figs 4, 5). A significant increase in *CPT* transcription was observed at mid-stages, concomitantly with the other genes involved in TAG storage (Figs 4, 5). Therefore, PC-DAG interconversion appears to be the major route for TAG enrichment in linoleic acid in the developing coffee endosperm.

### Acyl chain and DAG synthesis precedes formation of TAG and oil bodies in the developing endosperm

Even if lipid storage appeared to be a continuous process during the first 120 d of endosperm development (from stage 3 to stage 6, Fig. 1d), detailed analysis of the several pathways that contribute to TAG accumulation revealed two tightly coordinated sequential stages of transcription (Figs 4, 5). Plastidial genes involved in formation of acetyl-CoA, sequential addition of 2-carbon units, desaturation at the Δ_9_ position and, finally, FA release in the form of acyl-CoA all showed high levels of transcription at stage 3. Similarly, ER genes contributing to acylation on positions *sn*-1 and *sn*-2 of the glycerol (EL610524, DV681807, DV703295) were highly expressed from the onset of endosperm development. By contrast, genes for enzymes governing desaturation of 18:1–18:2 (Δ_12_ desaturase, EE196983), PC-DAG interconversion (CPT), elongation of very long acyl chains (KCS, FD528594) and acylation on *sn*-3 position of glycerol (DAGAT, DV711950), exhibited maximal gene transcription at stages 4–5, concomitantly with peak synthesis of the oil body proteins (oleosin, caleosin and steroleosin). There was therefore a striking delay of *c*. 30 d between the burst of gene expression related to the specific last steps of TAG assembly and storage and that related to FA and DAG biosynthesis.

### The singular fatty acid composition of the coffee endosperm could rely on high *FATb* transcription during lipid synthesis

The endosperm of the mature coffee seed exhibits a rather unusual seed fatty acid composition since it is rich in both palmitic (16:0) and linoleic (18:2) acids (i.e. *c*. 40%, Table S1). 16:0-ACP represents the first major branch point in FA biosynthesis since it is the substrate for two major activities, KASII, which initiates the biosynthesis of C18 FA, and FATb, which in turn triggers the entry of 16:0 in the ER acyl-CoA pool and, consequently, its incorporation in TAG (Fig. 5). High palmitate and linoleate content implies that neither *FATb* nor any of the genes encoding enzymes acting from 16:0-ACP to linoleoyl-PC are down-regulated during endosperm development. We observed that FATb as well as KASII, SAD, FaTA and Δ_12_ desaturase-encoding genes exhibited a pronounced and coordinated increase in transcription during the stage with the highest lipid synthesis rate (Figs 1d, 4). Consequently, one may hypothesize that this coordinated transcription of genes involved in the two branches starting from 16:0-ACP results in a balanced proportion of their respective end-products.

### Chlorogenic acids accumulate massively at the beginning of endosperm development only

Chlorogenic acids already represented 5% DM of the seed at stage 2, suggesting that these compounds are synthesized early in the perisperm tissue and are then loaded, probably via the apoplast, in the developing endosperm. Moreover, for the first time, we showed that CGA accumulate within a short period, at the beginning of endosperm development (between stages 3 and 4) (Fig. 6). This increase corresponded to a fourfold rise and total CGA reached almost 20% of DM at stage 4 (7% of DM in the mature seed) and was mostly explained by an abrupt rise in 5-caffeoyl quinate (5-CQA), which represented *c*. 82% of total CGA at stage 4 (69% in mature seeds) (Table S1). A rapid overview of expression profiles of phenylpropanoid-related genes suggests that the tight coordination of gene expression observed in the biosynthesis of CWP and lipids is not a characteristic shared by CGA metabolism. However, a more in-depth analysis of this complex pathway network enabled identification of various potential transcriptional control points for CGA synthesis in coffee. First, genes encoding PAL1, PAL2, C4H, two 4CL (AM117807 and EH662404) and three CCoAOMT (EL645414, DV682513 and DV703981) showed high expression levels at stages 3 or 4, when the endosperm was undergoing rapid development (Fig. 6). Expression of these genes therefore coincided with maximal CGA biosynthetic activity (Figs 1d, 6), suggesting they play a key role in their biosynthesis. Other genes encoding putative upstream enzymes such as 4CL (DV689729) and C3H (DQ269126) could also be related to CGA biosynthesis activity, as their expression at stage 3 was close to their maximal expression. Similarly, the bimodal expression pattern of *HCT1* suggests a dual role for this enzyme that could be directly involved in caffeoyl quinate anabolism. However, another key phenylpropanoid gene, *HQT*, did not show an expression profile that matches the CGA accumulation pattern, suggesting that this transcript is restricted to the perisperm and not directly involved in CGA accumulation in the endosperm.

### The phenylpropanoid transcriptional program switches from CGA to lignin biosynthesis during endosperm development

Chlorogenic acid biosynthetic activity ceased very early during endosperm development and was even followed by a drop in CGA relative content at stage 5 (Table S1 and Fig. 6). This drop was the result of the dilution of ACG in the growing endosperm as the absolute CGA content expressed on a seed basis remained stable (Fig. 1d). However, a slight decrease in absolute CGA content was still noticeable at stage 6 (Fig. 1d) and could be explained by the metabolic re-routing of these precursors towards lignin biosynthesis (Fig. 6). Searches were therefore conducted to identify genes potentially involved in CGA remobilization at late stages. Three *CCR* genes (DV689388, EH662414 and DV703229), two *F5H* genes (DV689190 and EH662405), *CCoAOMT1*, *HCT1* and one *CAD* gene (EE198746) exhibited maximal transcription levels at late stages. These genes encode enzymes that act downstream of caffeoyl quinate, supporting the hypothesis that the CGA pool serves as an intermediate in lignin biosynthesis during endosperm development. The high expression level of *HCT1* during late stages suggests the involvement of this enzyme in caffeoyl quinate catabolism. Moreover, a cationic peroxidase (POD) gene, encoding an enzyme which is directly involved in monolignol polymerization, displayed high induction and maximal expression at stage 5 during the process of endosperm hardening. Besides, one can hypothesize that genes corresponding to DV689190, DV703229 and EE198746 encode the F5H, CCR and CAD isoforms that are directly involved in the last steps of the monolignol biosynthesis (peak of expression at stage 5). It is also worth noting that none of the genes encoding enzymes involved in the first three steps of the phenylpropanoid pathway, namely PAL, C4H and 4CL, displayed an expression profile that matched the time window of the lignification process associated with endosperm hardening. This reinforces the hypothesis that the phenylpropanoid pathway is dissociated into two independent transcriptional events during seed development: first, CGA biosynthesis during the perisperm-endosperm transition; and second, CGA remobilization for monolignol biosynthesis during endosperm hardening.

## Discussion

**Fig. 7:**
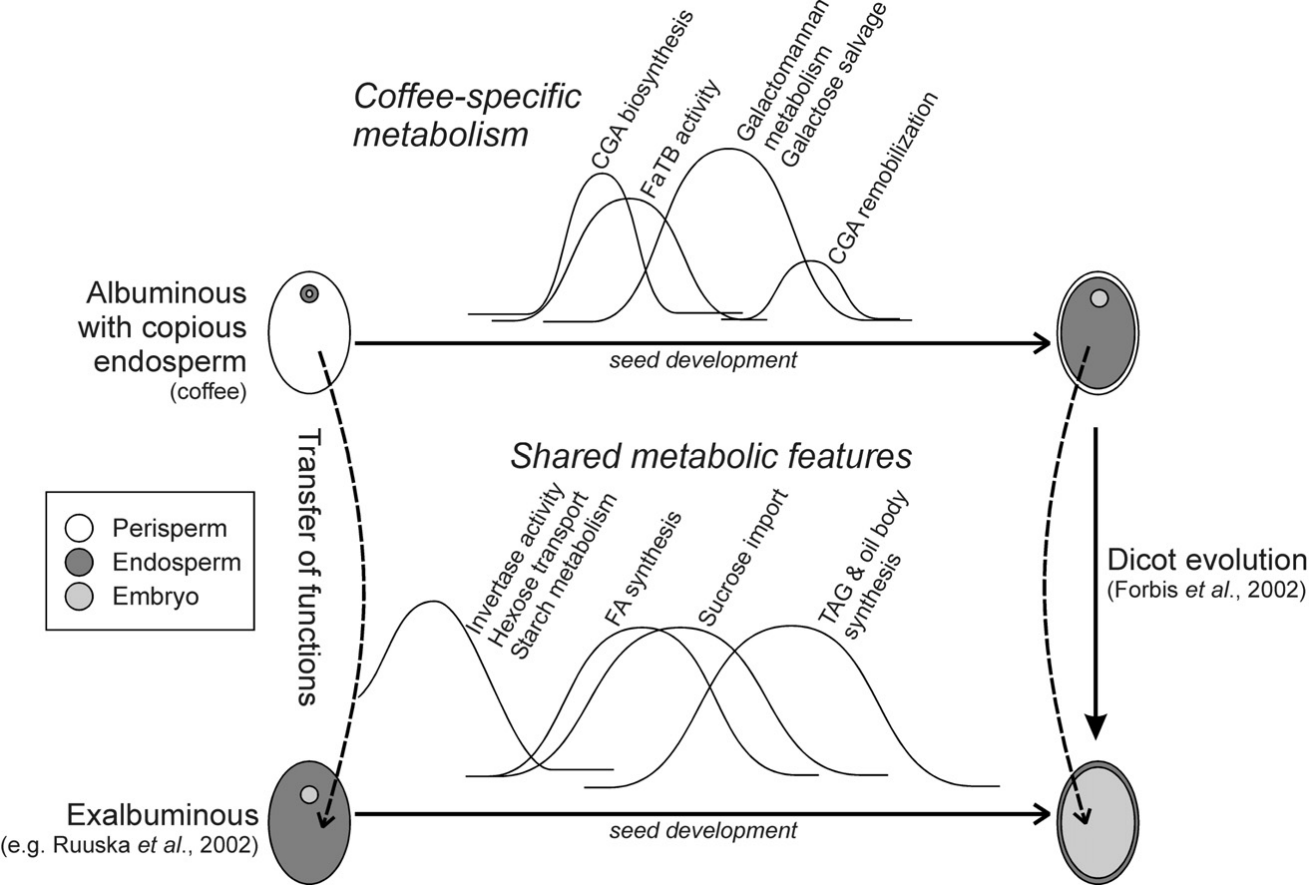
Simplified diagram showing the expression patterns of genes involved in storage-product synthesis in the developing coffee (*Coffea arabica*) seed. The upper part of the diagram highlights the temporal expression patterns of genes related to the biochemical specificities of the coffee seed, such as its very high chlorogenic acid (CGA) content, and its balanced palmitic : PUFA ratio. The lower part of the diagram highlights the developmental processes encountered both in the coffee seed and in exalbuminous seeds, as described in the literature. These shared metabolic features are further interpreted as reflecting a transfer of functions from perisperm to endosperm, and from endosperm to embryo in the course of dicot evolution.

The aim of the present work was to piece together the metabolic pathways of the major seed storage compounds in coffee, using the current genomic knowledge acquired on model plants. This approach provided new data likely to explain the peculiar traits of the mature coffee endosperm, such as its original FA composition, the transient accumulation of RFO and its exceptional CGA content (upper part of Fig. 7).

Indeed, the mature coffee seed presents the rather unusual trait that its storage lipids contain the same high concentrations of palmitic and linoleic acids (i.e. *c*. 40%). Out of the 7000 species for which seed oil FA composition was recorded on the SOFA database (Aitzetmuller*et al.*, 2003), only 38 contain more than 30% of both 16:0 and 18:2. This unusual fatty acid composition is also found in flowers of Arabidopsis (Dormann*et al.*, 2000) and, to a lesser extent, in cottonseed oil, which contain 25 and 50% palmitic and linoleic acids, respectively (Pirtle*et al.*, 1999). In contrast to other PUFA-rich seeds (e.g. *Brassica*, Arabidopsis, sunflower), we presume that the high 16:0 content of the coffee endosperm results from its high rate of *FATb* transcription during lipid synthesis. In the developing cotton embryo, high palmitoyl-ACP thioesterase activity has also been suggested to explain the high palmitic acid content of the cottonseed oil (Pirtle*et al.*, 1999). The *FATb* gene is not as highly expressed in the developing Arabidopsis embryo as it is in flower tissues (Dormann*et al.*, 2000; Schmid*et al.*, 2005). Even more convincingly, overexpression of *FATb* in embryos of Arabidopsis transgenic lines resulted in a palmitic : PUFA ratio identical to that of the coffee endosperm (i.e. 40 : 40%) (Dormann*et al.*, 2000).

We showed that the CGA seed content already represents 5% of the seed DM at stage 2, confirming that part of the biosynthetic activity occurs early in the perisperm tissues (Lepelley *et al.*, 2007). Our data also show that it rises abruptly at the beginning of the endosperm development, reaching *c*. 20% DM at stage 4. To our knowledge, such a high content has never been observed in any other plant, definitely raising *Coffea* seed as a model for the biosynthesis and accumulation of CGA. A better understanding of CGA metabolism in developing coffee seed may indeed serve other species whose seeds accumulate CGA in substantial amounts, either transiently, like tobacco (5% of DM at mid-stages, Sano*et al.*, 1982), or progressively throughout development, like sunflower (up to 4% of DM, Dorell, 1978). The present work enabled the identification of genes whose transcription is coordinately regulated during the very short phase of CGA accumulation. Among them, the transcript profile of enzymes that are represented by a single-copy gene may reveal important features in the genetic control of the phenylpropanoid pathway. Only one *C4H* gene has been described so far in Arabidopsis, although a genome-wide candidate gene annotation was performed. Its single-copy state assigns a central position to the *C4H* gene in the phenylpropanoid pathway, as its ubiquitous expression was confirmed in a variety of organs. Additionally, its supplemental induction was verified under a range of stress conditions (Bell-Lelong*et al.*, 1997; Raes*et al.*, 2003). Similarly, only one *C4H*transcript was found in *Coffea* ESTs, suggesting it also plays a major role in coffee. Interestingly, a pronounced transient peak of C4H-specific mRNA accumulation was observed at stages 3 and 4, the peak stage of CGA storage. In addition, it provided evidence at the transcriptional level that CGA are partly remobilized towards lignin synthesis during cell wall hardening. This result corroborates the negative correlation between CGA content and cell wall-bound phenolic polymers reported in developing *C. arabica* seedlings (Aerts & Baumann, 1994). Moreover, the time window for transcription of genes involved in CGA catabolism (between stages 4 and 5) corresponded to that of cell wall thickening as shown by the 40% increase in cell wall material (Table S1) and previous electronic microscopy observations (Dentan, 1985). Among genes involved in this metabolic re-routing, the accumulation of *HCT1* transcripts during late stages of seed development suggests the involvement of this enzyme in CQA catabolism. Interestingly, the recombinant HCT enzyme from *C. canephora* has recently been shown to catalyze 5-CQA hydrolysis (Lepelley *et al*., 2007) and could thus represent the first step in CGA mobilization.

Raffinose family oligosaccharides generally accumulate (2–10% DM) in the late stages of seed development, concomitantly with the acquisition of desiccation tolerance, and later serve as reserves that are rapidly mobilized during germination (Peterbauer & Richter, 2001). These physiological functions do not occur in coffee since only traces of RFO were detected in mature seeds. By contrast, nonnegligible amounts of RFO were transiently present during the storage phase and could be used as a reservoir for their primary components (i.e. sucrose and galactose). Our profiling data suggest that RFO remobilization was involved in the extensive demand for galactose for galactomman synthesis at mid-stages of development. This transient accumulation of RFO was closely associated with several genes linked to galactose metabolism that also peaked at mid-stages, such as galactinol synthase, the first enzyme of the RFO biosynthesis pathway, shown to be a crucial checkpoint in RFO synthesis and allocation in *Ajuga reptans* (Sprenger & Keller, 2000). Similarly, UDP-glucose 4′-epimerase was shown to control the structure and composition of galactose-containing CWP in *Arabidopsis thaliana* (Nguema-Ona*et al.*, 2006). We also provided evidence for a functional myo-inositol oxidation pathway during CWP biosynthesis in coffee. This pathway has recently been characterized at the molecular level in higher plants and is thought to play an important role in hemicellulosic polysaccharides biosynthesis (Kanter*et al.*, 2005).

In addition, the present work substantiates previous biochemical or histological studies of coffee seed development that did not include a transcript profiling effort comparable to that carried out here. For example, the dramatic increase in activity of the CWP biosynthetic machinery between stages 3 and 6 revealed here at the transcriptional level is in full agreement with biochemical data reported by Redgwell *et al.* (2003). It also perfectly matches the time window previously described for cell wall thickening, as determined by histological observations (Dentan, 1985). Moreover, the expression of genes involved in galactose-to-mannan branching, such as galactomannan galactosyltransferase and α-galactosidase (Marraccini*et al.*, 2005), appears to be concomitant (150 DAF) with the rapid decrease in the degree of substitution of the galactomannan observed at the biochemical level during *C. arabica* seed development (Redgwell *et al*., 2003). Regarding lipid synthesis, the burst of expression of genes involved in TAG assembly and storage observed here at mid-stages supports the observations by electronic microscopy of oil body accumulation in endosperm cells at 110–150 DAF (Dentan, 1985). Geromel*et al.* (2006) reported starch accumulation in the transient perisperm of coffee from histological observations. Our finding that transcripts encoding plastid enzymes involved in starch biosynthesis all accumulate in early developmental stages and then progressively decline during seed development fully corroborates these data. Finally, some of our transcriptional profiles also confirm patterns of enzymatic activity described elsewhere. For instance, the peak of expression of vacuolar invertase genes observed at early stages in the present study is highly consistent with the high vacuolar invertase enzyme activity previously detected in the perisperm (Geromel*et al.*, 2006).

As the present study was on an albuminous seed with copious endosperm, it also enabled comparison of temporal expression patterns in this category of seeds with those previously observed in model oily exalbuminous seeds (Ruuska*et al.*, 2002). This comparison revealed that the two seed types share important regulatory mechanisms for sugar and lipid accumulation, independent of the origin and ploidy level of the storage tissue (lower part of Fig. 7). For instance, our data suggest that acyl chain and DAG synthesis precedes formation of TAG and oil bodies. This result is in full agreement with the study of Ruuska *et al.* (2002), which described the storage metabolism in the Arabidopsis embryo using microarrays and revealed the existence of two different patterns of expression as the major finding arising from their lipid biosynthesis data. Indeed, though Arabidopsis embryo development is considerably shorter (*c*. 20 d), these authors demonstrated the existence of two categories of lipid-related genes that can be distinguished by a significant interval between the onset of transcription and peak transcription. Interestingly, in the developing Arabidopsis embryo, genes encoding FA synthesis enzymes, such as ACCase and KASI, are also expressed earlier than genes encoding oleosins, linoleate desaturase, and FA elongase. This important feature of the developing Arabidopsis embryo was confirmed here in a dicot albuminous seed species, to our knowledge for the first time.

Regarding sugar metabolism, we found that the increase in sucrose content at mid-stages is closely associated with the expression profile of sucrose transporters. Again, this developmental feature of the endosperm calls to mind the increase in sucrose content that occurs in the mid-stages of embryo development in some exalbuminous seeds (e.g. Arabidopsis; Baud*et al.*, 2002), or in seeds with few persisting cell layers of endosperm (e.g. tobacco; Tomlinson*et al.*, 2004). Similar transcriptional control for sucrose transporters has been seen in Arabidopsis (Baud*et al.*, 2005) and in barley, where their expression occurred in maternal as well as filial tissues, but was preferentially expressed in the cells of the nucellar projection and the endospermal transfer layer, which are the sites of sucrose exchange between the two tissues (Weschke*et al.*, 2000).

The early metabolic and transcriptomic events also bear a striking resemblance in the two seed categories, whereas the two transient tissues that predominate at these stages (perisperm and liquid endosperm, respectively) are of very different origin. The coffee perisperm is characterized by a high hexose-to-sucrose ratio (present data, Rogers*et al.*, 1999b; Geromel*et al.*, 2006; Privat*et al.*, 2008), which recalls the high amounts of hexoses in the transient endosperm of exalbuminous seeds (Borisjuk*et al.*, 1998; Baud*et al.*, 2002; Hill*et al.*, 2003). High invertase activity and the resulting high hexose-to-sucrose ratio have been shown to control the rate of cell division in the early stages of developing seeds (Wobus & Weber, 1999). We also showed that transcripts encoding plastid enzymes involved in starch biosynthesis accumulated transiently in the early stages of the coffee seed development. This phenomenon is similar to the transient accumulation and subsequent breakdown of starch in the early stages of embryo development in oily seed species such as rape and Arabidopsis (Eastmond & Rawsthorne, 2000; Ruuska*et al.*, 2002). Strikingly, a high H/S ratio has also been observed during the very first stage of coffee endosperm development when cell division predominates (Geromel*et al.*, 2006), suggesting that this ratio is a pertinent indicator of cell division, independent of the tissue origin.

We also observed that the perisperm and the endosperm use different transcriptional programs towards most of the ER lipid biosynthesis reactions. This notable divergence between the transient maternal tissue and the persistent triploid filial tissue warrants comparison with transcriptional specialization observed among tissues of other seed systems, such as the seed coat, the transient liquid endosperm, and the embryo in developing exalbuminous seed (Gallardo*et al.*, 2007), or the maternal pericarp, the filial embryo, and the endosperm in cereal caryopsis (Sreenivasulu *et al*., 2004, 2006; Neuberger*et al.*, 2008). For example, Neuberger *et al.* (2008) observed that most lipid biosynthesis enzymes were encoded by two paralog genes in the developing barley seed, one with preferential expression in the embryo and the other with preferential expression in the endosperm.

It is now accepted that a copious endosperm surrounding an underdeveloped embryo is a primitive character among seed plants and that evolution of dicots led to an increase in the embryo-to-endosperm ratio (Forbis*et al.*, 2002). These comparisons therefore suggest that during the course of evolution, a transfer of functions occurred between the two transient tissues (perisperm to endosperm), on the one hand, and between the two storage tissues (endosperm to embryo) on the other (Fig. 7). This transfer does not appear to have induced major disturbances in the coordination of the transcriptional program of seed filling.

## Supporting Information

**Table S1** Free sugar, chlorogenic acid and total lipid contents of cell wall material, and fatty acid composition of developing seeds of *C. arabica*

**Table S2** Composition of metabolic pathways and relative expression values of genes analyzed: Genbank accession of *Coffea* EST; protein Genbank accession for the best match in public databases (*BLASTX* results); E values for BLAST and putative functional identification

**Table S3** List of specific primers used for real-time RT-PCR

**Fig. S1** Separation of RT-PCR products on 4% (w/v) agarose gels revealed single products of the expected size for all reactions.
