# Supplementary material for: Metabolic pathways in tropical dicotyledonous albuminous seeds: Coffea arabica as a case study
Source: New Phytol. 2009 Apr;182(1):146–62. doi: 10.1111/j.1469-8137.2008.02742.x (PMC2713855; doi:10.1111/j.1469-8137.2008.02742.x)
Supplement: Supplementary file 1 [file nph0182-0146-SD1.doc]

**Supporting Information**

**Table S1** Cell wall material, free sugar, chlorogenic acid, and total lipid contents, and fatty acid composition of developing seeds of *C. arabica*

| **Developmental stage** | | **ST2** | **ST3** | **ST4** | **ST5** | **ST6** | **ST7** |
| --- | --- | --- | --- | --- | --- | --- | --- |
| **Cell-Wall material** | | 24.9^d^ | 32.7^c^ | 43.3^b^ | 60.7^a^ | 62.1^a^ | 61.6^a^ |
| **Sugars and sugar alcohols** | |  |  |  |  |  |  |
|  | **Total** | 31.03^a^ | 25.24^b^ | 9.15^c^ | 8.15^c^ | 7.78^c^ | 7.98^c^ |
|  | **Sorbitol** | 0 | 0 | 0.18 | 0.27 | 0.03 | 0 |
|  | **Glucose** | 16.51^a^ | 12.50^a^ | 0.45^b^ | 0.16^b^ | 0.21^b^ | 0.26^b^ |
|  | **Fructose** | 1.59^a^ | 1.45^a^ | 0.37^b^ | 0.06^b^ | 0.17^b^ | 0.08^b^ |
|  | **Sucrose** | 8.00 | 8.26 | 7.04 | 7.21 | 7.16 | 7.53 |
|  | **Raffinose** | 0.17^a^ | 0.03^b^ | 0.08^a^ | 0.09^a^ | 0.02^b^ | 0.01^b^ |
|  | **Stachyose** | 0.05 | 0 | 0.36 | 0.11 | 0 | 0 |
|  | **Myo-inositol** | 4.71^a^ | 3.00^b^ | 0.67^c^ | 0.25^c^ | 0.19^c^ | 0.10^c^ |
| **CGAs** | |  |  |  |  |  |  |
|  | **Total CGAs** | 5.21^d^ | 4.86^d^ | 19.73^a^ | 11.50^b^ | 6.71^c^ | 7.00^c^ |
|  | **3-CQA** | 0.05^c^ | 0.05^c^ | 0.05^c^ | 0.13^b^ | 0.22^a^ | 0.22^a^ |
|  | **4-CQA** | 0.07 | 0.07 | 0.41 | 0.57 | 0.48 | 0.51 |
|  | **5-CQA** | 3.49 | 3.13 | 16.13 | 8.93 | 4.58 | 4.83 |
|  | **3-FQA** | 0.02^a^ | 0.02^a^ | 0.00^b^ | 0.00^b^ | 0.00^b^ | 0.00^b^ |
|  | **4-FQA** | 0.04^d^ | 0.04^d^ | 0.26^a^ | 0.14^b^ | 0.06^cd^ | 0.08^c^ |
|  | **5-FQA** | 0.42^c^ | 0.38^c^ | 1.20^a^ | 0.64^b^ | 0.34^c^ | 0.32^c^ |
|  | **di3.4-CQA** | 0.05^c^ | 0.06^c^ | 0.09^bc^ | 0.10^b^ | 0.11^a^ | 0.11^a^ |
|  | **di3.5-CQA** | 0.98^b^ | 1.03^b^ | 1.51^a^ | 0.92^bc^ | 0.83^c^ | 0.85^c^ |
| **Lipids** |  |  |  |  |  |  |  |
|  | **Total lipids** | 14.14^c^ | 11.75^d^ | 19.30^a^ | 16.33^b^ | 13.50^c^ | 13.65^c^ |
| **Fatty acid composition** | |  |  |  |  |  |  |
|  | **Myristic (14:0)** | 0.266^a^ | 0.308^a^ | 0.193^b^ | 0.124^c^ | 0.124^c^ | 0.127^c^ |
|  | **Palmitic (16:0)** | 49.0^a^ | 51.4^a^ | 43.1^b^ | 39.7^b^ | 40.1^b^ | 41.1^b^ |
|  | **Palmitoleic (16:1)** | 0.461^a^ | 0.469^a^ | 0.114^b^ | 0.083^b^ | 0.037^b^ | 0.037^b^ |
|  | **Margaric (17:0)** | 0.201^ab^ | 0.223^a^ | 0.171^ab^ | 0.151^b^ | 0.136^b^ | 0.138^b^ |
|  | **Stearic (18:0)** | 7.36^a^ | 7.08^a^ | 4.21^d^ | 4.95^cd^ | 5.99^b^ | 5.56^bc^ |
|  | **Oleic (18:1)** | 5.01^b^ | 3.65^c^ | 3.14^c^ | 5.17^b^ | 6.63^a^ | 6.53^a^ |
|  | **Cis-Vaccenic (18:1-n7)** | 3.92^a^ | 3.50^b^ | 0.87^c^ | 0.17^d^ | 0.12^d^ | 0.00^d^ |
|  | **Linoleic (18:2)** | 22.9^b^ | 22.2^b^ | 42.9^a^ | 44.7^a^ | 42.6^a^ | 42.6^a^ |
|  | **Linolenic (18:3)** | 8.43^a^ | 9.01^a^ | 3.54^b^ | 2.63^c^ | 1.88^d^ | 1.76^d^ |
|  | **Arachidic (20:0)** | 1.80 | 1.55 | 1.36 | 1.77 | 1.76 | 1.55 |
|  | **Gondoic (20:1)** | 0.083^b^ | 0.067^b^ | 0.096^b^ | 0.159^a^ | 0.202^a^ | 0.197^a^ |
|  | **Behenic (22:0)** | 0.450 | 0.292 | 0.220 | 0.281 | 0.329 | 0.243 |
|  | **Lignoceric (24:0)** | 0.170 | 0 .095 | 0.096 | 0.127 | 0.082 | 0.099 |

Values are expressed as percentages of dry mass (% DM) except for fatty acids which are expressed as relative contents (% of total fatty acids). For a given class of compounds, values followed by the same letter were not significantly different at *P* ≤ 0.05 according to one-way ANOVA and post-hoc comparisons of means. CQA, caffeoyl quinic acid; FQA, feruloyl quinic acid.
