# Supplementary material for: Metabolic pathways in tropical dicotyledonous albuminous seeds: Coffea arabica as a case study
Source: New Phytol. 2009 Apr;182(1):146–62. doi: 10.1111/j.1469-8137.2008.02742.x (PMC2713855; doi:10.1111/j.1469-8137.2008.02742.x)
Supplement: Supplementary file 2 [file nph0182-0146-SD2.doc]

**Supporting Information – legend to Table S2**

**Table S2** Composition of metabolic pathways and relative expression values of genes analyzed: Genbank accession of coffea EST, protein Genbank accession for the best match in public databases (*BLASTX* results), E values for BLAST and putative functional identification. Effect of the developmental stage on real-time RT-PCR data: ANOVA results (F and *P*). Relative expression values of genes analyzed by real-time RT-PCR using the mean Ct of all genes under study as a reference. Fold change ratios were obtained from the equation (1+E)^-Δ(ΔCt)^ where:

E is the estimation of PCR efficiency,

ΔCt = Ct _target gene_ − Ct_mean of all genes_,

and Δ(ΔCt) = ΔCt _stage of interest_ − ΔCt_stage of maximal expression_.

The stage showing maximal expression was therefore normalized to 1. Fold change ratios compared to the reference and obtained from the equation (1+E)^-ΔCt^ are also presented.
