# Supplementary material for: Metabolic pathways in tropical dicotyledonous albuminous seeds: Coffea arabica as a case study
Source: New Phytol. 2009 Apr;182(1):146–62. doi: 10.1111/j.1469-8137.2008.02742.x (PMC2713855; doi:10.1111/j.1469-8137.2008.02742.x)
Supplement: Supplementary file 3 [file nph0182-0146-SD3.doc]

**Supporting Information**

**Table S3** List of specific primers used for real-time RT-PCR. PCR efficiencies were estimated for each primer pair using LinRegPCR Software (Ramaker*s et* al., 2003)

| Gene number | GeneBank Accession | Putative function | Primer F | Primer R | Product size | PCR Efficiency |
| --- | --- | --- | --- | --- | --- | --- |
| 1 | AF460203 | Phenylalanine ammonia-lyase 1 (Atpal2; At3g53260) | TGAAGCTCATGTCATCGACC | CCGTGTTTTTCACTGAGGCC | 96 | 1.97 |
| 2 | DV678076 | 4 Coumarate CoA ligase (At4cl2; At3g21240) | TTCCTTCAATCGGTCCACTATG | CGGAGGCAACTGAGAATACAA | 107 | 1.95 |
| 3 | Am117807 | 4 Coumarate CoA ligase (At4cl8; At3g48990) | tgagtttggatgacaggttcg | gattcggatgtcgctctttct | 82 | 1.88 |
| 4 | DV689729 | 4 Coumarate CoA ligase (At4cl1; At1g51680) | ATTTGGGTTCTCTCCGTCAAC | CGGGACTACTGGACTGCCTAA | 91 | 1.98 |
| 5 | FD528592 | 4 Coumarate CoA ligase (At4cl6; At4g05160) | ccagccctgcttatctatggt | tgcagaacctcttccaccttt | 118 | 1.88 |
| 6 | DV691155 | 4 Coumarate CoA ligase (At4cl9; At1g20510) | GCACCGTCCTCAAACTACTCA | GCCGTGCAAAAATACAGAGC | 118 | 1.93 |
| 7 | DQ269127 | Coumaroyl 3'-hydroxylase C3H2 | tcgagaaaagagcggaggag | aaaggaggccaatgatggtg | 101 | 1.91 |
| 8 | DV682513 | Caffeoyl-CoA 3-O-methyltransferase (Atccoaomt7; At4g26220) | GGGCAGTAAGCAGTAGGGAGT | TGATTGCCATGCTCTTGAAAC | 87 | 1.87 |
| 9 | DV689803 | Caffeoyl-CoA 3-O-methyltransferase (Atccoaomt4; At3g62000) | ATACTTGTGCTTACGCGCTCA | TGATAATGTCCTCTGGCATGG | 117 | 1.87 |
| 10 | DV703981 | Caffeoyl-CoA 3-O-methyltransferase (Atcoaomt6; At1g67980) | TCTCCACACCAGCTTTCTTGA | CTGCTCTTGCACTTCCTGATG | 102 | 1.96 |
| 11 | DV689190 | Ferulate 5 hydroxylase (At5h1; At4g36220) | TTCATTACTCGGGGGTTTTTC | TTTGGTGGACTTGCTTACTGC | 93 | 1.89 |
| 12 | DV703229 | Cinnamoyl CoA reductase (Atccr1; At1g15950) | GGGAAGCAGTGTGGAAAACC | GATGATGCGAAGAATGGTCA | 139 | 1.85 |
| 13 | DV704414 | Cinnamoyl CoA reductase (At2g23910) | TAAGCATTCGGTCCATAGCC | GCAGGGAAAAGAAGTTGTGGT | 84 | 1.85 |
| 14 | EE197082 | Cinnamoyl CoA reductase (Atccr-like3; At2g33590) | TATTTCTCATCGTCGGGGTTT | GTTACAGGAGCAGGGGGATAC | 104 | 1.93 |
| 15 | DV710279 | Cinnamoyl CoA reductase (Atccr-like5; At5g58490) | TGCTTCGTCTCTTTCTCATCC | GCTACATTGGGTCTTGGTTGG | 97 | 1.92 |
| 16 | EE198746 | Cinnamyl-alcohol dehydrogenase (Atcad8; At4g37990) | GGGAAGCGAATGATAAAGTGC | TTGTCCGAAAGTGGTGTTGAC | 111 | 1.94 |
| 17 | EE194808 | Cinnamyl-alcohol dehydrogenase (Atcad2 ; At2g21730) | CAACTCCAACTCGATCACCAA | ATGGGGGTTCACAAATTACCC | 110 | 1.80 |
| 18 | DV708968 | Cinnamyl-alcohol dehydrogenase (Atcad5; At4g34230) | TATGGCAAATCCCACAGCATA | GAGCTTGGAAGTGGAGAAGGA | 140 | 1.86 |
| 19 | DV695361 | Cinnamyl-alcohol dehydrogenase (Atcad1; At1g72680) | CCCATCAAAAGTGAGGACTGG | TTGGAGACCATGTTGGAGTTG | 113 | 1.92 |
| 20 | EE198213 | Plastidial Homomeric Acetyl-CoA Carboxylase (At1g36180 ) | CCACGCTCAGGGATTGATT | TCTGGTGCTCGTATTGGTGTC | 83 | 1.75 |
| 21 | DV691687 | Acetyl-CoA carboxylase, a-Carboxyltransferase (At2g38040) | CAAGAGGTTCGGGGATGATT | TGTGCTGCGATACTGTGGAA | 121 | 1.74 |
| 22 | CF588828 | Acetyl-CoA carboxylase, b-Carboxyltransferase (AtCg00500) | TGCCGTTTAGTTGGCCTATG | TCATTCGGAGGAGGAACCTT | 101 | 1.98 |
| 23 | DV688205 | Acetyl-CoA carboxylase, Biotin Carboxyl Carrier Protein (At5g16390) | CCATCCACAGCAACCTCATT | CCACCATTCTCACTCCAAGG | 135 | 1.88 |
| ~~24~~ | ~~DV690489~~ | ~~Hydroxyacyl-ACP dehydrase~~ | ~~TGCTCCTCTCTGTGCTTTGAG~~ | ~~TCCCACCTTGTTTTCCTCGT~~ | ~~105~~ | ~~1.83~~ |
| 25 | EE199224 | Enoyl ACP reductase | GCAAAAACTGAGGGAATGGT | TCAGCCACACCAGCAATAAA | 116 | 1.94 |
| 26 | DV674569 | Plastidial Enoyl-ACP Reductase | CAGCGCGACTTCTTAGTGGA | TACAAGGGTGCTGGCTTTTG | 86 | 1.92 |
| 27 | EE191863 | Stearoyl ACP desaturase, SAD | ATGGCTTTGCAGTTGAATGC | TTGAAGCCATGATGAATTTGG | 106 | 1.93 |
| 81 | DV698082 | Plastidial Pyruvate Dehydrogenase E1a (At1g01090) | TTGTCAGGTGCTATGAGGTTGG | GCAAATCCATCCGTTGAAAAC | 121 | 2.00 |
| 28 | DV705646 | Plastidial Pyruvate Dehydrogenase | AGCCTCTTGGGTCAGCAAAT | CTGGCGAGTGAAGCAGAGTT | 139 | 1.87 |
| 29 | DV682450 | Fatty acid desaturase, FAD6 | TCAGGGTGAAACTGCCAACT | AGGAACCTTGGAGGAATGGA | 135 | 1.90 |
| 30 | DV692820 | Glycerol-Phosphate Acyltransferase (GPAT8; AT4G00400) | GACTCTTCAGGGGCGATTTC | CAGGAGAACTGGGGCAAGTA | 134 | 1.82 |
| 31 | DV691014 | Phosphatidate Phosphatase (PAP; At1g15080) | GGTGCAGTCAAGACCAGGAA | ACCTTGCTTAGAGGGCGAAA | 98 | 1.78 |
| 32 | DV698726 | Phosphatidate Phosphatase (PAP'; At1g15080) | CAGCAAAGGACCCAGAAGTG | TTGGCGATGTTTCCCTGAT | 134 | 1.78 |
| 33 | DV703295 | Diacylglycerol Cholinephosphotransferase (CPT; At3g25585) | CCAGAATGGCGTGATGAAGA | TGTTCCTGTATGGGCTGTGC | 110 | 1.88 |
| 34 | DV675072 | Acylglycerol-phosphate acyltransferase (LPAT putative; AT2G27090) | GCCGTTGGACACAAAGACAT | AGGCATTGTTGGTGCTGAGT | 140 | 1.92 |
| 35 | DV681807 | Acylglycerol-phosphate acyltransferase (LPAT2; AT3G57650) | GCATCCACCCCTTAGACAACAT | GGCAAGAAATGAGCAACTTCAG | 138 | 1.96 |
| 36 | DV678913 | Beta-ketoacyl-CoA synthase, KCS | AACAGGCAACCCAGATGATG | GCTTGAACGGCTAAGGGATT | 124 | 1.92 |
| 37 | DV697042 | Beta-ketoacyl-CoA synthase, KCS | TCGTCCTCGTCCAGTTTACC | TTGCGAATGAGCCAACCAT | 113 | 1.98 |
| 38 | FD528594 | Long chain Acyl-CoA synthetase (LACS1; At2g47240) | GTTTCGGATGGGAGGTGTAG | CCATGTGTGTTCGATAGAGATGTT | 98 | 1.88 |
| 39 | DV697121 | Long chain Acyl-CoA synthetase (LACS2; At1g49430) | TCTGTCATGGAAGTGAAGCAA | GGACCAGGAGCTGTTGATTTT | 140 | 1.75 |
| 40 | DV707808 | Long chain Acyl-CoA synthetase (LACS3; At1g64400) | TGGCGGAACCAATTTTAAGAG | AGAATGCCAAGAATCGAATGC | 117 | 1.88 |
| 41 | DV692589 | Long chain Acyl-CoA synthetase (LACS8; At2g04350) | TTCAACTGCTTGCTTCTTTGG | CAGGGCGAGTATGTAGCTGTG | 138 | 1.95 |
| 42 | DV698296 | Long chain Acyl-CoA synthetase (LACS9; At1g77590) | GCTTCCTTTGCTACCTTGGAA | TCGAGAAATGGGCTGAAGATT | 115 | 1.95 |
| 43 | DV683183 | Diacylglycerol acyltransferase (DAGAT at2g19450) | CTCCCCACCAACTTCCATTTA | TGTTCGCAAGAAGGTAGATGC | 101 | 1.90 |
| 44 | DV711950 | Diacylglycerol acyltransferase (DAGAT at3g51520) | CAGCACACACATCGGTTGAC | GGTTCCCCTGGTTGTATTGA | 115 | 1.97 |
| 45 | DV697030 | Phospholipid:diacylglycerol acyltransferase (PDAT; AT5G13640) | TAAGTTCCGTCCCCATTTGC | CATCGTCTTGGCTTCCTTCA | 118 | 1.89 |
| ~~46~~ | ~~FD528593~~ | ~~Phospholipid Diacylglycerol acyltransferase (PDAT; AT5G13640)~~ | ~~TGTGGGAAGGGCATCATTGT~~ | ~~TCAGCCCAACACAAAGGTCT~~ | ~~97~~ | ~~1.98~~ |
| ~~47~~ | ~~DV707770~~ | ~~Sterol 24-C methyltransferase~~ | ~~TAAAGCAGGCACCGTCTCAT~~ | ~~AGTCCCCTTCCTCAGAATGC~~ | ~~125~~ | ~~1.94~~ |
| 48 | EL610521 | Sucrose transporter | GCACCCAATCTGGAGGAAG | AATTCGGTGGTGACCCATTC | 92 | 1.87 |
| 49 | DV676158 | Sucrose Transporter (SUT1 ou SUT4) | CAGTGTTCCCTATGCCTTGATA | CGCTTCCAAGAGAGACCAAAAT | 122 | 1.99 |
| 50 | CF589106 | Monosaccharide transporter AtSTP1 (At1g11260) | aaatccccaaaagctggacata | accaccgctatcacaaccttct | 140 | 1.95 |
| 51 | DV667384 | Polyol transporter | CTTCTTTGGGGGATTTGTCTTG | CAGTGCTGCTTCCAGACCATAT | 108 | 1.86 |
| 52 | EE199674 | Aldose 1-epimerase | CTCTTGTGGGGTCAGCCATT | ATTAAAAAGGCCCCGACGAA | 103 | 1.98 |
| 53 | DV702921 | Rafinose synthase | CGTGAAAGATAAGGCCAATCC | CTTCCGCTTCCATTATTACGC | 143 | 1.92 |
| 54 | EE194479 | Phytase | GTTCCATTCCCTCCATCCAG | TGGCAAGACGAGCTTTTTGA | 127 | 1.89 |
| 55 | EL610522 | Alpha-galactosidase precursor, gal1 | CGTTGGGATTACTGGGGAAG | AGGCTGCAAATGTCCGATTC | 115 | 2.00 |
| 56 | AJ877911 | Beta galactosidase | CTCGCAGGAGCCTTTTAGCA | GCAGCAAGCCCCTTTGATAC | 139 | 2.00 |
| 57 | EE196065 | Cellulose synthase catalytic subunit | TTGCCAAGGCATCCTAGAAC | TCTTCTCCCAAAACAAGACGTC | 110 | 1.91 |
| 58 | FD528595 | Beta-mannosidase | ATTGGCCGTGGTGGTAATGT | TGCCATCATCCTCTTGACCA | 112 | 1.96 |
| 59 | DV699694 | Aconitase | AGGAAGGTGCTCCAAAGCAT | GCATGGCACAAGATGAGGTT | 93 | 1.96 |
| 60 | EE193323 | Fructose-bisphosphate aldolase, cytoplasmic | ATTTTGAGGGGCGTGTTCAT | ACAGTTCCAGCGAGGGCATA | 88 | 2.00 |
| 61 | EE200490 | Fructose-bisphosphate aldolase, plastidial | CCCCAATGAACCATCTCAGC | ATAGGCACCAGGCCATTCTG | 93 | 1.88 |
| 62 | EE195441 | Hexokinase | CCACAGTCGATGGCAAGAAG | TGTTTGAGCCAGCAAGAGGA | 102 | 1.60 |
| 63 | DV698259 | GAUT (Galacturonosyltransferase) : Pectin synthesis | CGATACAACCACCCAGATGTT | CTGATTTAGGCAGCAGACCA | 115 | 1.60 |
| 64 | EE198504 | Phosphoglucomutase, cytosolic | ACGATCAAACCGATGAAAGC | GATATTGTGGTGCGGAAGGA | 108 | 1.94 |
| 65 | EE196728 | UDP glucose pyrophosphorylase | GGCAATCCTTCTGCAATCAA | CATGGAAAATGGTGGACCTG | 96 | 1.95 |
| 66 | EE195881 | ADP glucose pyrophosphorylase (plastid) | GGCACCACAACCTCATCTGT | CAACCTCATCGCTCGCTATC | 87 | 1.90 |
| 67 | DV711473 | Phosphoglucose isomerase (G6P isomerase) | AAATCGCCGTCAGTTCCAG | CGAGCAGGAGTTTGAGTGGT | 132 | 1.94 |
| 68 | DV710699 | Phosphofructokinase | ggggtgcaagtttttccaat | gttgggattgttgagcgtgt | 95 | 1.95 |
| 69 | DV704130 | Fructokinase | CTGCACCAGGACCATCAAAT | TGGAAAACCTGCTCTGCATC | 140 | 1.84 |
| 70 | DV698417 | Phosphoglucomutase, plastidial | AATGAAGAACGATGCCCGTTAT | GCGATGGAGGGAGTACAAAAAG | 105 | 1.89 |
| 71 | DV677099 | UDP-glucose 6-dehydrogenase (EC-Number 1.1.1.22 ) | TGGAAATCTTATGGATGCTGGA | AGCCAAGCTAATACAGCCCAAA | 111 | 1.86 |
| 72 | DV666345 | UDP-glucuronate 4-epimerase AtUGlcAE1 (EC 5.1.3.6) | TGTAGTTTGGGATGCCTACGAG | GCTGGTTTCTGCATGTTGTCAT | 120 | 2.00 |
| 73 | EE196005 | Mannose 1 P guanyltransferase | TCCAGTTCTGTTTACGGGTTGA | ATAGCTTCACCGGACTTCTTGG | 107 | 1.88 |
| 74 | DV691631 | Galactomannan galactosyltransferase | CGTTTGAGGCCACTGACACTAA | CAGCATCACTTCTGGCTGGTAG | 147 | 1.83 |
| 75 | DV684998 | Raffinose like – alkaline alpha galactosidase | GGGAGAGGAGATTGAGGAGGAG | TGACACCCCGTGAAGTGAGT | 139 | 1.89 |
| 76 | DV666551 | Alcaline glucosidase (raffinose as substrate) | CAGAGAAGCGAGACGCAATAAA | AGGGCAAACACTTGAACTCCAT | 132 | 1.99 |
| 77 | DV712985 | Stachyose synthase putative | GGTTCTGTTCAAGCCAGTGATG | CACCTTTTGGTAGCCGCATTA | 118 | 1.89 |
| 78 | DV689723 | Galactosyltransferase | CGAAGAGGCCAAAGAAGTTGAT | TCCAATTCCCATAACTCAGAAGC | 100 | 1.72 |
| 79 | DV697147 | Galactinol synthase golS-1 | TTCCTCGCTGTATTTGTCTCCA | TAGGGTCGTGGGTTCGATTTAC | 148 | 1.97 |
| 80 | DV683570 | Acyl-ACP Thioesterase Fat A | TGGGATATTTACGACGACGAGA | AATGTAGCGAGCAGGTCTAGGC | 126 | 2.00 |
| 81 | DV678710 | MIOX1 Myo-inositol oxygenase | GTGCGCTTTTGACGAATCAA | TGTCCAGTCCACAACCTTCG | 116 | 1.92 |
