# Supplementary material for: Metabolic pathways in tropical dicotyledonous albuminous seeds: Coffea arabica as a case study
Source: New Phytol. 2009 Apr;182(1):146–62. doi: 10.1111/j.1469-8137.2008.02742.x (PMC2713855; doi:10.1111/j.1469-8137.2008.02742.x)
Supplement: Supplementary file 4 [file nph0182-0146-SD4.ppt]

## Slide 1
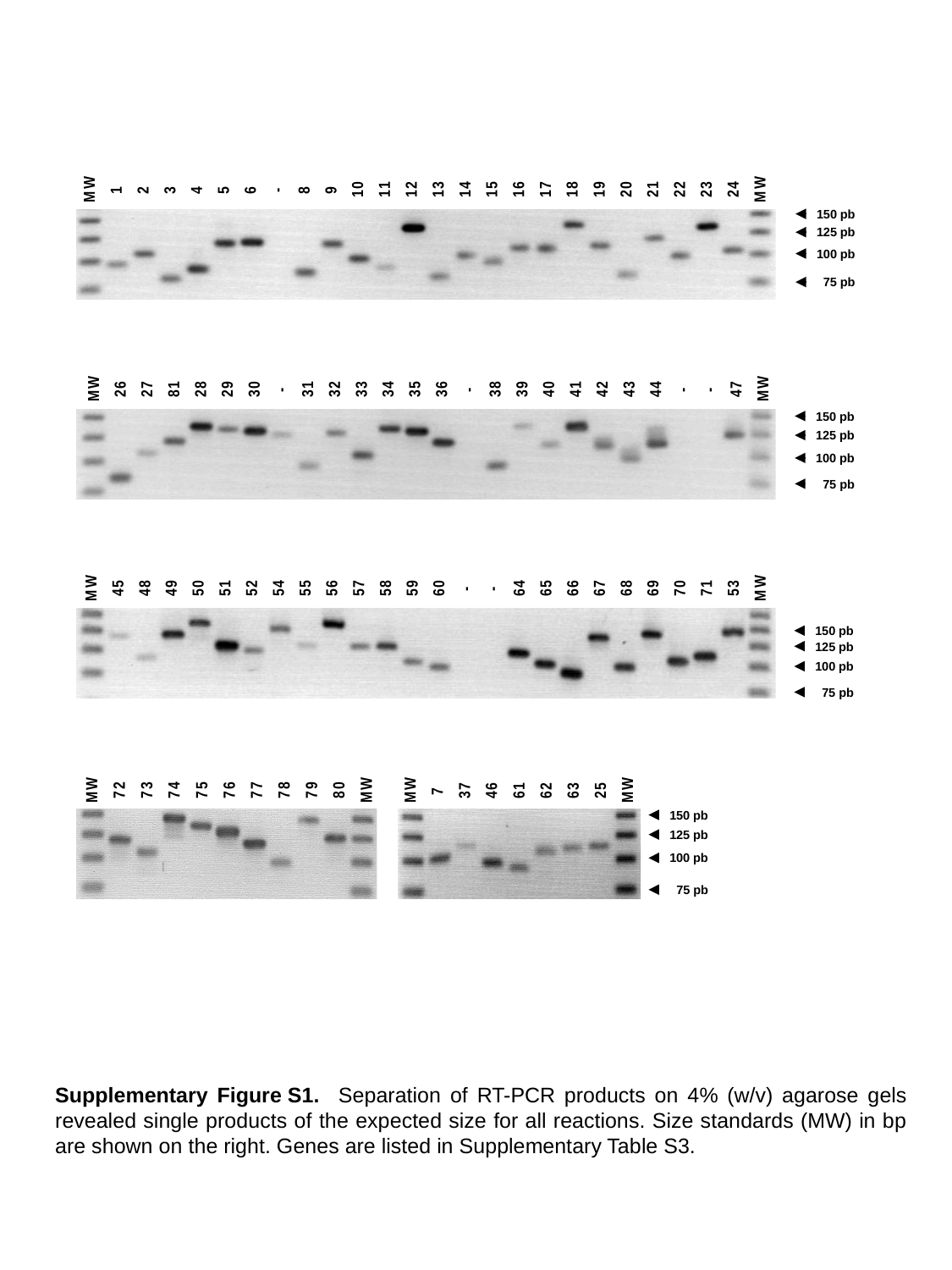

150 pb
125 pb
100 pb
 75 pb
150 pb
125 pb
100 pb
 75 pb
150 pb
125 pb
100 pb
 75 pb
150 pb
125 pb
100 pb
 75 pb
Supplementary Figure S1. Separation of RT-PCR products on 4% (w/v) agarose gels revealed single products of the expected size for all reactions. Size standards (MW) in bp are shown on the right. Genes are listed in Supplementary Table S3.
